# Long-term real-world outcomes of first-line immunotherapy in non-small cell lung cancer – a population-based cohort study in Sweden

**DOI:** 10.2340/1651-226X.2025.42746

**Published:** 2025-03-17

**Authors:** Gunnar Wagenius, Anders Vikström, Anders Berglund, Stina Salomonsson, Goran Bencina, Xiaohan Hu, Diana Chirovsky, Hans Brunnström

**Affiliations:** a Department of Oncology-Pathology, Karolinska Institute, Stockholm, Sweden; b Theme Cancer, Medical Unit Head and Neck, Lung, and Skin Tumors, Thoracic Oncology Center, Karolinska University Hospital, Stockholm, Sweden; c Department of Respiratory Medicine, Linköping University Hospital, Linköping, Sweden; d Epistat AB, Uppsala, Sweden; e MSD, Value and Implementation Outcomes Research, Stockholm, Sweden; f MSD, Value and Implementation Outcomes Research, Madrid, Spain; g Value and Implementation Outcomes Research, Merck & Co., Inc., Rahway, NJ, USA; h Division of Pathology, Department of Clinical Sciences Lund, Lund University, Lund, Sweden; i Department of Genetics, Pathology, and Molecular Diagnostics, Skåne University Hospital, Lund, Sweden

**Keywords:** Combination therapy, immune checkpoint inhibitor, monotherapy, overall survival, PD-L1

## Abstract

**Background:**

In a previous study, we explored real-world programmed death-ligand 1 (PD-L1) testing and treatment patterns for patients with advanced non-small cell lung cancer (NSCLC) in the era of immune-oncology. The present study aimed to investigate overall survival (OS) with PD-(L)1 inhibitors with longer-term follow-up in the Swedish setting.

**Materials and methods:**

Data were extracted from the Swedish National Lung Cancer Registry for patients with NSCLC stage IIIB-IV and ECOG performance status (PS) 0–2 who initiated first-line systemic treatment from 1-April-2017 to 30-June-2021 with data cut-off 30-June-2022. OS and Kaplan–Meier estimates were calculated from start of the PD-(L)1 inhibitor therapy, with subgroups based on nonsquamous/squamous (NSQ/SQ) histology, and further by PS, and PD-L1 status (available from 1-January-2018) provided sufficient sample size.

**Results:**

We identified 784 (NSQ:590/SQ:194) patients treated with first-line PD-(L)1 inhibitor monotherapy and 369 (NSQ:305/SQ:64) patients receiving combination regimens. Median OS (95% confidence interval [CI]) was 15.2 (12.4–17.7) and 12.9 (10.6–15.8) months with monotherapy and 17.0 (13.6–23.9) and 18.0 (13.9-NA) months with combination regimens for NSQ/SQ patients. In PS2, median OS with monotherapy was 5.0 (3.7–7.1) and 8.9 (6.2–12.9) months for NSQ/SQ patients (*n* = 138/59), 5.3 (3.6–13.4) months with combination regimens in NSQ (*n* = 58) and not evaluable in SQ patients. For PS0-1 patients with tumor cell PD-L1 expression ≥50%, the median OS for NSQ was 23.8 (17.7–29.3) and 27.3 (21.6-NA) months for monotherapy/combination therapy (*n* = 281/55), while the median OS for combination regimens for PD-L1 <1% and 1–49% was 18.6 (12.1–26.9) and 15.9 (10.8–26.7) months (NSQ; *n* = 65/87).

**Interpretation:**

Real-world OS in Swedish patients receiving first-line PD-(L)1 inhibitor-based regimens was consistent with that observed in clinical trials. Moderate OS rates were observed in PS2, with limited sample sizes. Further research is needed in these patients, as well as in high PD-L1, given the slightly longer OS for combination therapy compared to monotherapy seen for NSQ.

## Introduction

Lung cancer is the most common malignancy and the leading cause of cancer-related death worldwide [1]. In Sweden, it is the fifth most common tumor, but still the leading cause of death due to cancer [2]. Non-small cell lung cancer (NSCLC) accounts for approximately 85% of all lung cancer, and the majority of lung cancer patients are diagnosed with the disease in an advanced stage (IIIB/C or IV) [3, 4].

As in other countries, the treatment landscape of advanced NSCLC has changed dramatically in Sweden since the introduction of frontline immunotherapies in 2017. In a previous study, we reported an increasing trend in the use of first-line programmed death receptor-1/programmed death-ligand 1 (PD-(L)1) inhibitor-based regimens for patients with advanced NSCLC from 2017 to 2020 [5]. We also reported the associated real-world outcomes by treatment class and histology but with limited follow‑up.

Sweden has a tax-financed healthcare system that provides a uniform system for cancer diagnostics and treatment across geographic areas, socioeconomic and age groups. The Swedish National Lung Cancer Registry (NLCR) is a population-based quality database that contains details on demographics, diagnostic procedures including testing results and overall survival (OS) for 98% of Swedish lung cancer patients, which allows for comprehensive analysis of a real-world setting.

In the present study, with additional patients included and 12 months of additional follow-up compared to our previous study [5], the aim was to further investigate treatment outcomes with a focus on OS in patients treated with first-line PD-(L)1 inhibitor-based regimens in Sweden.

## Material and methods

The Swedish NLCR is a Clinical Quality Register, certified by the Swedish Association of Local Authorities and Regions, and includes lung cancer patients diagnosed in 2002 and later with a completeness of 98% compared to the National Swedish Cancer Register, to which reporting is mandatory by the Swedish law. The NLCR includes detailed information on demographic and clinical characteristics such as date of diagnosis, performance status (PS), histopathological diagnosis, stage and biomarker status. Data on PD-L1 testing and results were available in the registry from 1-January-2018.

In addition, information was also extracted from the Individual Patient-Overview Lung Cancer (IPO), which is a part of the NLCR. The IPO was created by an interdisciplinary team involving physicians and nurses from different hospitals in Sweden together with patient representatives. The aim was to create user-friendly decision support by collecting longitudinal clinically important information for each patient with lung cancer presented in an interactive graphical display, trying to overcome gaps in Electronic Healthcare Records. The IPO includes detailed information on the follow-up of the patient with regard to surgical procedures, radiation therapy, systemic medical treatments including treatment duration, reasons for discontinuation and adverse event reporting, and real-world outcomes.

In the present study, patients aged 18 years and older diagnosed with locally advanced or metastatic (stage IIIB-IV) NSCLC disease with a record of first-line treatment from 1-April-2017, which is the date that the first PD-(L)1 inhibitor was recommended to use as first-line therapy for advanced NSCLC (PD-L1 expression ≥50%) in Sweden, to 30-June-2021 were identified in the NLCR and in the IPO. The data cut-off for the present analysis was 30-June-2022 to ensure a theoretical minimum follow-up of 1 year (time from treatment initiation to data cut-off date). Exclusion criteria included patients with an Eastern Cooperative Oncology Group (ECOG) PS higher than 2 or unknown PS at index treatment initiation, patients enrolled in clinical trials and patients treated with chemo-radiation with curative intent. The study population was divided into a nonsquamous (NSQ) and a squamous (SQ) cohort, with further descriptive subgroup analyses reported by ECOG PS (0–1 and 2) and by PD-L1 expression (<1%, 1–49% and ≥50%) based on sufficient sample size. The study was conducted following the guidelines of the Declaration of Helsinki and was approved by the Swedish Ethical Review Authority (DNR 2020-01547).

## Statistical methods

Demographic characteristics, biomarker testing and class of therapy were described by histology groups (NSQ, SQ) using descriptive statistics. In the second step, to understand real-world outcomes with PD-(L)1 inhibitors, the analysis was restricted to only subjects with at least one record of PD-(L)1 inhibitor treatment in combination or as monotherapy. Demographic and clinical characteristics and biomarker testing were then summarized by histology groups for the overall population, and by ECOG PS. Continuous variables were described by medians and interquartile ranges (IQR) and categorical variables were reported as numbers and percentages. The time-to-event approach was used to study OS using the Kaplan–Meier methods for patients receiving PD-(L)1 inhibitor monotherapy and PD-(L)1 inhibitor combination, by histology and in the subgroups defined by ECOG PS and PD-L1 expression; patients with positive EGFR, ALK or ROS1 test were excluded to align with the regulatory approved indications. OS was defined from the start date of first-line treatment until the date of death, or the last date of follow-up (30-June-2022), whichever came first. In addition, reasons for discontinuation of PD-(L)1 inhibitors by histology groups and ECOG PS were presented. No statistical tests were applied, and the statistical analyses were performed using R version 4.1.2.

## Results

A total of 4,082 subjects at least 18 years old at diagnosis were identified with a histologically or cytologically confirmed NSCLC diagnosis in the Swedish NLCR and at least one record of a first-line treatment between 1-April-2017 and 30-June-2022. Subjects with stage IA–IIIA disease at diagnosis or at the start of first-line treatment (*n* = 219), ECOG PS 3 (*n* = 138) or 4 (*n* = 12) or missing (*n* = 64), patients enrolled into clinical trials (*n* = 208) or with no information on enrolment (*n* = 23) or who were put on chemo-radiation therapy with curative intent (*n* = 266) were excluded. The remaining 3,119 patients with a confirmed diagnosis of unresectable stage IIIB/C or IV NSCLC with first-line treatment and PS0-2 consisted of 2,515 (81%) NSQ and 604 (19%) SQ patients. Of these, 305 and 590 NSQ patients were treated with first-line PD-(L)1 inhibitors in combination and monotherapy, respectively, and were included for further analyses. The corresponding numbers for SQ histology were 64 and 194 patients, respectively.

Demographic, clinical and biomarker characteristics of patients treated with a first-line PD‑(L)1 inhibitor monotherapy and PD-(L)1 inhibitor combination regimen by histology and ECOG PS are summarized in Tables 1 and 2 for NSQ and SQ, respectively. The sample size of ECOG 2 patients was limited within the histology and treatment subgroups defined. Among patients with NSQ histology, 138 (23.4%) and 58 (19.0%) patients receiving PD‑(L)1 inhibitor monotherapy and combination regimens had ECOG PS2, respectively. Among patients with SQ histology, 59 (30.4%) and 14 (21.9%) patients receiving PD‑(L)1 inhibitor monotherapy and combination regimens had ECOG PS2, respectively. ECOG 2 patients tended to be older with a higher proportion with stage IV disease relative to the overall populations receiving PD‑(L)1 inhibitor monotherapy and combination regimens defined by histology.

**Table 1:** Descriptive characteristics by treatment groups and performance status in nonsquamous patients.

	PD-(L)1 inhibitor combination			PD-(L)1 inhibitor monotherapy		
	Total	ECOG PS 0-1	ECOG PS 2	Total	ECOG PS 0-1	ECOG PS 2
**All subjects**	305 (100.0)	247 (100.0)	58 (100.0)	590 (100.0)	452 (100.0)	138 (100.0)
**Sex**						
Male	131 (43.0)	103 (41.7)	28 (48.3)	248 (42.0)	199 (44.0)	49 (35.5)
Female	174 (57.0)	144 (58.3)	30 (51.7)	342 (58.0)	253 (56.0)	89 (64.5)
**Age**						
Mean (SD)	67.95 (8.87)	67.43 (8.48)	70.21 (10.11)	70.25 (8.47)	69.70 (8.89)	72.07 (6.62)
Median [IQR]	69.0 [64.0, 74.0]	68.0 [63.0, 73.0]	73.0 [65.0, 76.0]	72.0 [66.0, 76.0]	71.5 [65.8, 75.0]	73.0 [68.0, 76.8]
**Smoking status**						
Current smoker	99 (32.5)	85 (34.4)	14 (24.1)	207 (35.1)	155 (34.3)	52 (37.7)
Former smoker	163 (53.4)	125 (50.6)	38 (65.5)	330 (55.9)	249 (55.1)	81 (58.7)
Never smoker	43 (14.1)	37 (15.0)	6 (10.3)	53 (9.0)	48 (10.6)	5 (3.6)
**ECOG performance status**						
ECOG PS 0-1	247 (81.0)	247 (100.0)	0 (0.0)	452 (76.6)	452 (100.0)	0 (0.0)
ECOG PS 2	58 (19.0)	0 (0.0)	58 (100.0)	138 (23.4)	0 (0.0)	138 (100.0)
**Stage**						
IIIB	21 (6.9)	19 (7.7)	2 (3.4)	62 (10.5)	48 (10.6)	14 (10.1)
IIIC	11 (3.6)	10 (4.0)	1 (1.7)	18 (3.1)	14 (3.1)	4 (2.9)
IV	273 (89.5)	218 (88.3)	55 (94.8)	510 (86.4)	390 (86.3)	120 (87.0)
**Histology**						
Adenocarcinoma	273 (89.5)	223 (90.3)	50 (86.2)	533 (90.3)	411 (90.9)	122 (88.4)
Non-small cell carcinoma unspecified/other	32 (10.5)	24 (9.7)	8 (13.8)	57 (9.7)	41 (9.1)	16 (11.6)
**PD-L1 testing**	279 (91.5)	226 (91.5)	53 (91.4)	487 (82.5)	370 (81.9)	117 (84.8)
**PD-L1 expression**						
<1% (negative)	80 (28.7)	65 (28.8)	15 (28.3)	55 (11.3)	45 (12.2)	10 (8.5)
1%-49%	112 (40.1)	87 (38.5)	25 (47.2)	57 (11.7)	40 (10.8)	17 (14.5)
50%+	66 (23.7)	55 (24.3)	11 (20.8)	366 (75.2)	281 (75.9)	85 (72.6)
Undetermined/no results	21 (7.5)	19 (8.4)	2 (3.8)	9 (1.8)	4 (1.1)	5 (4.3)
**EGFR testing**	278 (91.1)	225 (91.1)	53 (91.4)	542 (91.9)	416 (92.0)	126 (91.3)
**EGFR positive**	7 (2.5)	7 (3.1)	0 (0.0)	14 (2.6)	9 (2.2)	5 (4.0)
**ALK testing**	274 (89.8)	222 (89.9)	52 (89.7)	525 (89.0)	405 (89.6)	120 (87.0)
**ALK positive**	2 (0.7)	1 (0.5)	1 (1.9)	5 (1.0)	4 (1.0)	1 (0.8)
**ROS1 testing**	213 (69.8)	171 (69.2)	42 (72.4)	417 (70.7)	323 (71.5)	94 (68.1)
**ROS1 positive**	2 (0.9)	1 (0.6)	1 (2.4)	5 (1.2)	5 (1.5)	0 (0.0)

ECOG: Eastern Cooperative Oncology Group; IQR: interquartile range; PD-L1: programmed death ligand 1; PS: performance status; SD: standard deviation.

**Table 2:** Descriptive characteristics by treatment groups and performance status in squamous patients.

	PD-(L)1 inhibitor combination			PD-(L)1 inhibitor monotherapy		
	Total	ECOG PS 0-1	ECOG PS 2	Total	ECOG PS 0-1	ECOG PS 2
**All subjects**	64 (100.0)	50 (100.0)	14 (100.0)	194 (100.0)	135 (100.0)	59 (100.0)
**Sex**						
Male	38 (59.4)	30 (60.0)	8 (57.1)	117 (60.3)	86 (63.7)	31 (52.5)
Female	26 (40.6)	20 (40.0)	6 (42.9)	77 (39.7)	49 (36.3)	28 (47.5)
**Age**						
Mean (SD)	68.27 (7.58)	67.80 (7.28)	69.93 (8.64)	71.78 (8.16)	70.81 (8.62)	74.00 (6.54)
Median [IQR]	70.0 [64.0, 74.0]	69.0 [64.0, 73.0]	72.0 [68.3, 75.8]	72.5 [67.0, 78.0]	71.0 [66.0, 77.5]	74.0 [70.5, 78.5]
**Smoking status**						
Current smoker	23 (35.9)	17 (34.0)	6 (42.9)	79 (40.7)	55 (40.7)	24 (40.7)
Former smoker	38 (59.4)	30 (60.0)	8 (57.1)	103 (53.1)	70 (51.9)	33 (55.9)
Never smoker	3 (4.7)	3 (6.0)	0 (0.0)	12 (6.2)	10 (7.4)	2 (3.4)
**ECOG performance status**						
ECOG PS 0-1	50 (78.1)	50 (100.0)	0 (0.0)	135 (69.6)	135 (100.0)	0 (0.0)
ECOG PS 2	14 (21.9)	0 (0.0)	14 (100.0)	59 (30.4)	0 (0.0)	59 (100.0)
**Stage**						
IIIB	7 (10.9)	5 (10.0)	2 (14.3)	41 (21.1)	28 (20.7)	13 (22.0)
IIIC	9 (14.1)	5 (10.0)	4 (28.6)	22 (11.3)	12 (8.9)	10 (16.9)
IV	48 (75.0)	40 (80.0)	8 (57.1)	131 (67.5)	95 (70.4)	36 (61.0)
**PD-L1 testing**	61 (95.3)	47 (94.0)	14 (100.0)	152 (78.4)	104 (77.0)	48 (81.4)
**PD-L1 expression**						
<1% (negative)	16 (26.2)	10 (21.3)	6 (42.9)	19 (12.5)	16 (15.4)	3 (6.2)
1%-49%	33 (54.1)	27 (57.4)	6 (42.9)	31 (20.4)	19 (18.3)	12 (25.0)
50%+	10 (16.4)	8 (17.0)	2 (14.3)	100 (65.8)	67 (64.4)	33 (68.8)
Undetermined/no results	2 (3.2)	2 (4.2)	0 (0.0)	2 (1.4)	2 (2.0)	0 (0.0)
**EGFR testing**	53 (82.8)	41 (82.0)	12 (85.7)	121 (62.7)	80 (59.7)	41 (69.5)
**EGFR positive**	1 (1.9)	1 (2.4)	0 (0.0)	0 (0.0)	0 (0.0)	0 (0.0)
**ALK testing**	54 (84.4)	41 (82.0)	13 (92.9)	123 (63.4)	80 (59.3)	43 (72.9)
**ALK positive**	1 (1.9)	1 (2.4)	0 (0.0)	0 (0.0)	0 (0.0)	0 (0.0)
**ROS1 testing**	45 (70.3)	33 (66.0)	12 (85.7)	106 (54.6)	69 (51.1)	37 (62.7)
**ROS1 positive**	0 (0.0)	0 (0.0)	0 (0.0)	0 (0.0)	0 (0.0)	0 (0.0)

ECOG: Eastern Cooperative Oncology Group; IQR: interquartile range; PD-L1: programmed death ligand 1; PS: performance status; SD: standard deviation.

**Figure 1:**
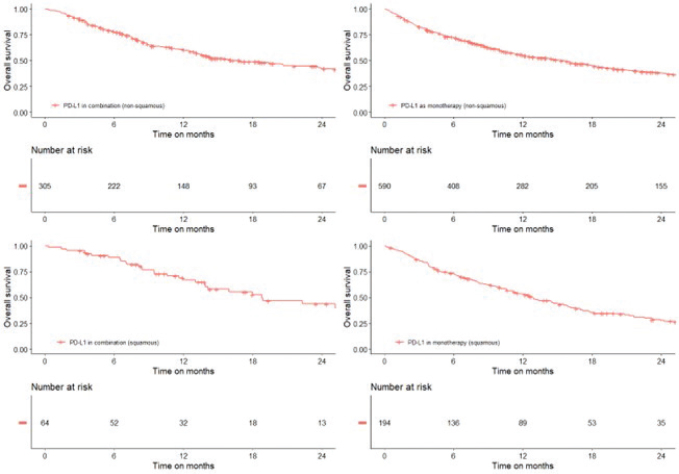
Overall survival for PD-(L)1 inhibitors in combination and as monotherapy by histology (nonsquamous, sqaumous) in the overall population with ECOG performance status (PS) 0-2.

Median theoretical follow-up, defined as the time from treatment initiation to data cut-off (30-June-2022), was 26.7 months (IQR: 13.6–40.9 months) in patients with NSQ histology and 18.8 months (IQR: 9.9–33.2 months) in patients with SQ histology. Among patients initiating a first-line PD-(L)1 inhibitor monotherapy in the overall population with ECOG PS0-2, the median OS was 15.2 and 12.9 months for patients with NSQ and SQ advanced NSCLC, respectively (Figure 1; Table 3). As evident from the same figure and table, the median OS was 17.0 and 18.8 months for patients with NSQ and SQ, respectively, for combination therapy in the overall population.

**Table 3:** Overall survival for first-line PD-(L)1 treatment in combination and as monotherapy by histology (nonsquamous, squamous), performance status and PD-L1 expression.

	PD-(L)1 inhibitor combination			PD-(L)1 inhibitor monotherapy		
	Overall	ECOG PS 0-1	ECOG PS 2	Overall	ECOG PS 0-1	ECOG PS 2
**Nonsquamous**						
Patients (events)	305 (166)	247 (124)	58 (42)	590 (382)	452 (262)	138 (120)
**OS rates**						
Median, months (95% CI)	17.0 (13.6–23.9)	20.6 (15.9–26.9)	5.3 (3.6–13.4)	15.2 (12.4–17.7)	19.8 (17.6–24.4)	5.0 (3.7–7.1)
12 months	60.5	64.9	41.9	55.0	64.2	25.5
24 months	42.0	45.4	29.1	38.0	45.9	13.1
**Squamous**						
Patients (events)	64 (30)	50 (23)	14 (NR)	194 (134)	135 (90)	59 (44)
**OS rates**						
Median, months (95% CI)	18.8 (13.9–NA)	18.9 (14.1–NA)	NR	12.9 (10.6–15.8)	15.0 (11.6–17.2)	8.9 (6.2–12.9)
12 months	66.8	71.3	NR	53.1	57.8	42.6
24 months	43.5	44.6	NR	27.7	30.6	21.1

	PD-(L)1 inhibitor combination (ECOG PS 0-1)			PD-(L)1 inhibitor monotherapy (ECOG PS 0-1)		
	PD-L1 <1%	PD-L1 1-49%	PD-L1 ≥50%	PD-L1 <1%	PD-L1 1-49%	PD-L1 ≥50%

**Nonsquamous**						
Patients (events)	65 (36)	87 (46)	55 (22)	45 (NR)	40 (NR)	281 (158)
**OS rates**						
Median, months (95% CI)	18.6 (12.1–26.9)	15.9 (10.8–26.7)	27.3 (21.6–NA)	NR	NR	23.8 (17.7–29.3)
12 months	63.2	55.3	78.7	NR	NR	64.2
24 months	38.0	39.3	56.6	NR	NR	49.5
**Squamous**						
Patients (events)	10 (NR)	27 (NR)	8 (NR)	16 (NR)	19 (NR)	67 (47)
**OS rates**						
Median, months (95% CI)	NR	NR	NR	NR	NR	13.3 (10.6–18.1)
12 months	NR	NR	NR	NR	NR	57.1
24 months	NR	NR	NR	NR	NR	27.2

ECOG: Eastern Cooperative Oncology Group; NA: not available; NR: not reported; OS: overall survival; PD-L1: programmed death ligand 1; PS: performance status.

In the small subgroups of patients with ECOG PS2, the median OS in patients with NSQ histology was 5.3 and 5.0 months for PD-(L)1 inhibitor in combination and as monotherapy, respectively (Table 3). In comparison, among patients with ECOG PS2 and SQ histology, the median OS was 8.9 months for monotherapy while the combination therapy subgroup was too small for analysis. As expected, a higher survival rate was observed in patients with ECOG PS0-1 relative to patients with ECOG PS2 across the subgroups defined by the PD-(L)1 inhibitor in combination and as monotherapy and histology (Supplementary Figure 1).

Testing of PD-L1 expression (available in the registry from 1-January-2018, thus not registered for all patients with treatment/survival data in the cohort) in the tumor cells was recorded for >90% of the patients receiving combination immunotherapy and for approximately 80% of the patients treated with monotherapy (Tables 1 and 2). In Table 3, survival data for PS0-1 patients are presented with subgroups depending on PD-L1 expression status. In patients with NSQ histology receiving PD-(L)1 inhibitor combination therapy, there was a trend for increased OS with high PD-L1 expression: PD-L1 <1%, 18.6; PD-L1 1–49%, 15.9; PD-L1 ≥50%, 27.3 months. As expected, the majority of patients receiving PD-(L)1 inhibitor monotherapy exhibited high (≥50%) PD-L1 expression (Tables 1 and 2). In these patients, the median OS was 23.8 and 13.3 months in the NSQ and SQ histology groups, respectively (Table 3).

Reasons for discontinuation of PD-(L)1 inhibitors by treatment modality, histology and PS are found in Table 4. As evident from the table, discontinuation according to plan was more common for combination therapy, and disease progression was more common for monotherapy. In patients with ECOG PS2, reasons for discontinuation related to death and ‘other reasons’ were more common compared to PS0-1.

**Table 4:** Reason for discontinuation of first-line PD-(L)1 treatment in combination and as monotherapy by histology (nonsquamous, squamous) and performance status.

	PD-(L)1 inhibitor combination,			PD-(L)1 inhibitor monotherapy,		
	Nonsquamous patients			Nonsquamous patients		
	Overall (*n* = 305)	ECOG PS 0-1 (*n* = 247)	ECOG PS 2 (*n* = 58)	Overall (*n* = 590)	ECOG PS 0-1 (*n* = 452)	ECOG PS 2 (*n* = 138)
**Total patients with reason recorded, *n* (%)**	252 (82.6)	198 (80.2)	54 (93.1)	451 (76.4)	326 (72.1)	125 (90.6)
**Reason for discontinuation, *n* (%)**						
According to plan	137 (54.4)	124 (62.6)	13 (24.1)	39 (8.6)	34 (10.4)	5 (4.0)
Disease progression	26 (10.3)	17 (8.6)	9 (16.7)	192 (42.6)	151 (46.3)	41 (32.8)
Adverse events	37 (14.7)	26 (13.1)	11 (20.4)	80 (17.7)	63 (19.3)	17 (13.6)
Patients wish	3 (1.2)	1 (0.5)	2 (3.7)	5 (1.1)	1 (0.3)	4 (3.2)
Death	12 (4.8)	5 (2.5)	7 (13.0)	53 (11.8)	22 (6.7)	31 (24.8)
Other	37 (14.7)	25 (12.6)	12 (22.2)	82 (18.2)	55 (16.9)	27 (21.6)

	PD-(L)1 inhibitor combination,			PD-(L)1 inhibitor monotherapy,		
	Squamous patients			Squamous patients		
	Overall(*n* = 64)	ECOG PS 0-1(*n* = 50)	ECOG PS 2(*n* = 14)	Overall(*n* = 194)	ECOG PS 0-1(*n* = 135)	ECOG PS 2(*n* = 59)

**Total patients with reason recorded, *n* (%)**	51 (79.7)	40 (80.0)	11 (78.6)	148 (76.3)	96 (71.1)	52 (88.1)
**Reason for discontinuation, *n* (%)**						
According to plan	36 (70.6)	28 (70.0)	8 (72.7)	13 (8.8)	8 (8.3)	5 (9.6)
Disease progression	1 (2.0)	1 (2.5)	0 (0.0)	62 (41.9)	49 (51.0)	13 (25.0)
Adverse events	7 (13.7)	5 (12.5)	2 (18.2)	27 (18.2)	15 (15.6)	12 (23.1)
Patients wish	1 (2.0)	1 (2.5)	0 (0.0)	4 (2.7)	2 (2.1)	2 (3.8)
Death	1 (2.0)	1 (2.5)	0 (0.0)	20 (13.5)	10 (10.4)	10 (19.2)
Other	5 (9.8)	4 (10.0)	1 (9.1)	22 (14.9)	12 (12.5)	10 (19.2)

ECOG: Eastern Cooperative Oncology Group; PD-L1: programmed death ligand 1; PS: performance status.

## Discussion

In this population-based Swedish register study evaluating real-world outcomes of first-line PD-(L)1 inhibitor treatment in advanced/metastatic NSCLC, we included more patients and had a longer follow-up compared to our previous investigation [5], allowing for conclusive survival analyses in the overall study populations defined by histology, and descriptive subgroup analyses by ECOG PS and PD-L1 expression.

In our study, the median OS for monotherapy-treated PS0-1 patients was 23.8 months for NSQ and 13.3 months for SQ NSCLC with tumor cell PD-L1 expression ≥50% (Table 3), which is generally in line with pivotal PD-(L)1 inhibitor clinical trials. The median OS for first-line pembrolizumab monotherapy in PS0-1 advanced/metastatic NSCLC (mixed adenocarcinomas and squamous cell carcinomas) with PD-L1 expression ≥50% was 26.3 months in the KEYNOTE-024 study and 20.0 months in the KEYNOTE-042 study [6, 7]. Correspondingly, in our study, the median OS was 20.6 and 18.9 months for first-line PD-(L)1 inhibitor combination therapy in PS0-1 NSQ and SQ NSCLC (any PD-L1 expression). In clinical trials on NSQ NSCLC with comparable study populations, the median OS was 22.0 months for pembrolizumab–chemotherapy combination in the KEYNOTE-189 study [8] and 19.0–19.5 months for atezolizumab–chemotherapy combination in the IMpower150 study [9]. For SQ NSCLC, the median OS was 17.7 and 14.2 months, respectively, in the KEYNOTE-407 and IMpower131 studies [10, 11].

In our study, some patients treated with PD-(L)1 monotherapy exhibited negative or low tumor cell PD-L1 expression. This may at least partly be explained by adverse effects or contraindications for chemotherapy (and hence its exclusion) in patients planned for PD-(L)1 combination therapy, but further reasons could not be investigated in the present study. Thus, the subgroups with low/negative PD-L1 and monotherapy are expected to be skewed and we did not specifically analyze survival in these limited populations.

For patients treated with PD-(L)1 combination therapy a significant number exhibited high PD-L1 expression. We could calculate survival rates for NSQ cases in our material, with a median OS (for PS0-1) of 18.6 months for PD-L1 <1%, 15.9 months for 1–49% and 27.3 months for ≥50% (Table 3). The trend in longer OS for high PD-L1 tumors for PD-(L)1 inhibitor combination therapy is in line with recent European and US real-world studies. Verschueren et al. reported 10.0 months for PD-L1 <1%, 19.9 months for 1–49% and 26.2 months for ≥50% [12], while Waterhouse et al. reported 10.2 months for PD-L1 <1%, 11.8 months for 1–49% and 19.1 months for ≥50% NSQ NSCLC [13]. Both these studies included some PS > 1 cases, while Liu et al., restricted to PS0-1, reported a median OS of 13.2 months for PD-L1 <1%, 16.9 months for 1-49% and 20.6 months for ≥50% [14]. In contrast, Leonetti et al. reported a slightly shorter OS for PD-L1 ≥50% compared to the negative and low positive groups, but the proportion of tumors with high PD-L1 was very low in that study [15].

It is noteworthy that the descriptive survival rates appeared slightly higher in our study for PD-(L)1 combination therapy relative to monotherapy for PS0-1 NSQ with PD-L1 ≥50%, acknowledging the small sample size of 55 patients receiving combination therapy in this subgroup. Some factors may guide the choice of therapy for PD-(L)1 inhibitors, which are not feasible to measure in an observational database and are beyond the scope of this study, which may have influenced these findings. The combination therapy subgroup with high PD-L1 likely contained more patients with higher disease burden where a rapid treatment effect is desired. As previously mentioned, patients who receive PD-(L)1 inhibitor monotherapy may have co-morbidities, such as poor renal function, that may render them ineligible for platinum-based combination therapy. However, for most of the included years, PD-(L)1 inhibitor monotherapy was recommended in Sweden in advanced NSCLC with high PD-L1 expression, and it is unlikely that a significant part of the PS0-1 patients with high PD-L1 expression in our study was planned for combination therapy but were unable to receive chemotherapy.

A higher tumor response rate for PD-(L)1 inhibitor combination therapy than monotherapy in NSCLC with PD-L1 expression ≥50% has been suggested [16] essentially based on comparisons of KEYNOTE-024 and 042 with KEYNOTE-189 and 407 [6–8, 10]. However, there are also other real-world studies in line with these and our results. Dudnik et al. showed a median OS for PD-(L)1 combination of 20.4 months compared to 12.5 months for monotherapy in NSCLC with PD-L1 ≥50% [17]. Waterhouse et al. reported a median OS of 19.1 months for combination therapy and 15.3 months for monotherapy for NSQ NSCLC with PD-L1 ≥50%, while the numbers were 12.3 versus 11.9 months for SQ NSCLC [13]. To our knowledge, there is no prospective trial comparing monotherapy and combination therapy in NSCLC with high PD-L1 expression, which would be of interest to further investigate if a better survival for combination therapy applies to all relevant subgroups, and if there are patient characteristics that may further guide choice of therapy. Despite the lack of such studies, changes in treatment selection can already be seen. For example in the current Swedish clinical guidelines, combination therapy is recommended for PS0-1 patients with advanced NSCLC without actionable molecular alterations regardless of PD-L1 expression level, while monotherapy remains an alternative in high PD-L1 expression [18].

The patients with squamous cell carcinoma were too few in our material for investigation of survival rates in PD-L1 expression subgroups. In the whole SQ population, the longer median OC for combination therapy compared to monotherapy in the PS0-1 patients (Table 3) is noteworthy, but given the moderate difference (18.9 vs. 15.0 months) and the limitations of our study, strong conclusions cannot be drawn.

Acknowledging the small size of patients with ECOG PS2 in our study, the median OS for PS2 patients was moderate in our study, approximately 5 months for NSQ NSCLC for both monotherapy and combination therapy, and 8.9 months for monotherapy-treated SQ NSCLC, with generally wide confidence intervals observed (Table 3). To our knowledge, the PePS2 study is the only prospective study on PS2 immunotherapy-treated NSCLC patients. In that study, the median OS was 9.8 months with pembrolizumab in the first or subsequent line of treatment [19]. There is a limited number of large observational studies reporting on immunotherapy-treated PS2 NSCLC patients in the literature, with a broad range in median OS observed. Waterhouse et al. reported survival rates for PS≥2 based on the Flatiron Health oncology database. The median OS was 5.2 and 6.3 months for monotherapy and combination therapy, respectively, for NSQ NSCLC and 5.3 and 8.0 months for SQ NSCLC [13]. Veluswamy et al., also using data on PS≥2 patients from the Flatiron Health oncology database, reported a median OS of 7.1 months for monotherapy in tumors with PD-L1 expression ≥50% and 5.6 months for monotherapy and 7.9 months for combination therapy for PD-L1 <50% (all histologies) [20]. Facchinetti et al. reported a shorter survival for PS2 patients with PD-L1 ≥50% (all histologies), with a median OS of just 3.0 months [21]. Ahmed et al. reported a median OS of 8.3 months for PS2 while Meyers et al. reported a median OS of 3.3 months for PS ≥ 2 (all histologies), but both these studies included immunotherapy in any line of treatment and both monotherapy and combination therapy [22, 23]. As ECOG PS2 patients are under-represented in clinical trials, with a broad range in median OS observed in observational studies, further research is needed in these patients, preferably in the form of randomized controlled trials to assess the actual benefit of immunotherapy.

The strengths of the present study include the high degree of coverage of patients with equal access to healthcare and advanced diagnostics, detailed information on patient characteristics such as PS and PD-L1 testing, and complete death information. The main weakness is the retrospective registry approach with the lack of detailed information on treatment decisions and the lower coverage in the follow-up part of the registry (IPO), which could have led to potential selection bias. Also, the overall sample size and the follow-up time were somewhat limited for conducting subgroup analysis and prognostic models, for example, for SQ NSCLC.

## Conclusions

The real-world OS estimates for advanced NSCLC observed in Swedish patients receiving first-line PD-(L)1 inhibitor-based therapy were similar to what has been reported in pivotal clinical trials. Moderate OS rates were observed in patients with ECOG PS2, with limitations on small sample size. As ECOG PS2 patients are under-represented in clinical trials, with a broad range in median OS observed in observational studies, further research is needed in these patients. The slightly longer OS for combination therapy compared to monotherapy in NSQ NSCLC with PD-L1 ≥50% suggests the need for further investigations of optimal treatment in advanced NSCLC with high PD-L1 expression in prospective trials, and exploration of patient characteristics that may guide the choice of therapy.

## Supplementary Material

Long-term real-world outcomes of first-line immunotherapy in non-small cell lung cancer – a population-based cohort study in Sweden

## Data Availability

Data not available due to ethical restrictions.
