# Supplementary material for: Long-term real-world outcomes of first-line immunotherapy in non-small cell lung cancer – a population-based cohort study in Sweden
Source: Acta Oncol. 2025 Mar 17;64:42746. doi: 10.2340/1651-226X.2025.42746 (PMC11931854; doi:10.2340/1651-226X.2025.42746)

Supplementary material has been published as submitted. It has not been copyedited, or typeset by Acta Oncologica

Supplementary Figure 1. Overall survival for first-line PD-(L)1 treatment in combination and as monotherapy by histology (nonsquamous, squamous) and ECOG performance status (PS)

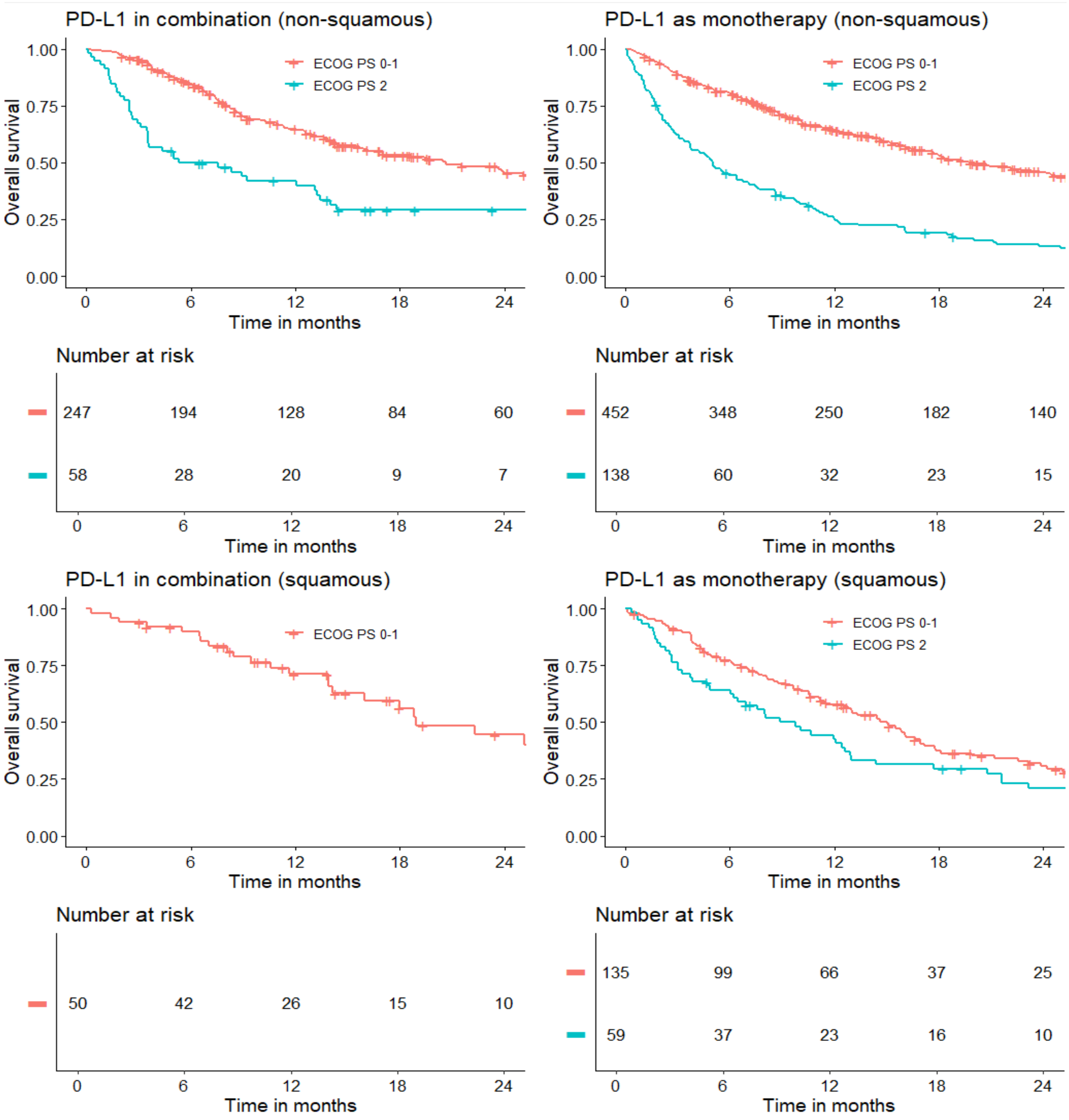

Supplement: Long-term real-world outcomes of first-line immunotherapy in non-small cell lung cancer – a population-based cohort study in Sweden [file AO-64-42746-s1.pdf]
